# Transcription-Based Prediction of Response to IFNβ Using Supervised Computational Methods

**DOI:** 10.1371/journal.pbio.0030002

**Published:** 2004-12-28

**Authors:** Sergio E Baranzini, Parvin Mousavi, Jordi Rio, Stacy J Caillier, Althea Stillman, Pablo Villoslada, Matthew M Wyatt, Manuel Comabella, Larry D Greller, Roland Somogyi, Xavier Montalban, Jorge R Oksenberg

**Affiliations:** **1**Department of Neurology, School of MedicineUniversity of California, San Francisco, CaliforniaUnited States of America; **2**School of Computing, Queen's UniversityKingston, OntarioCanada; **3**Department of Neuroimmunology, Hospital Vall d'HebronBarcelonaSpain; **4**Department of Neurology, Clinica Universitaria de Navarra, University of NavarraSpain; **5**Biosystemix, SydenhamOntarioCanada; European Bioinformatics InstituteUnited Kingdom

## Abstract

Changes in cellular functions in response to drug therapy are mediated by specific transcriptional profiles resulting from the induction or repression in the activity of a number of genes, thereby modifying the preexisting gene activity pattern of the drug-targeted cell(s). Recombinant human interferon beta (rIFNβ) is routinely used to control exacerbations in multiple sclerosis patients with only partial success, mainly because of adverse effects and a relatively large proportion of nonresponders. We applied advanced data-mining and predictive modeling tools to a longitudinal 70-gene expression dataset generated by kinetic reverse-transcription PCR from 52 multiple sclerosis patients treated with rIFNβ to discover higher-order predictive patterns associated with treatment outcome and to define the molecular footprint that rIFNβ engraves on peripheral blood mononuclear cells. We identified nine sets of gene triplets whose expression, when tested before the initiation of therapy, can predict the response to interferon beta with up to 86% accuracy. In addition, time-series analysis revealed potential key players involved in a good or poor response to interferon beta. Statistical testing of a random outcome class and tolerance to noise was carried out to establish the robustness of the predictive models. Large-scale kinetic reverse-transcription PCR, coupled with advanced data-mining efforts, can effectively reveal preexisting and drug-induced gene expression signatures associated with therapeutic effects.

## Abstract 2

By studying gene expression in patients with multiple sclerosis before and after therapy with beta interferon, it is possible to identify gene expression signatures that are associated with therapeutic effects

## Introduction

Interferons are small, inducible proteins secreted by nucleated cells in response to viral infection and other stimuli. They act in a paracrine fashion on other cells in their immediate vicinity, triggering a state of growth arrest, so that infected cells cannot be forced to produce viral proteins, and activating the process of programmed cell death, so that infected cells can be removed [1]. Interferons are important not only in the defense against a wide range of viruses but also in the regulation of immune responses and hematopoietic cell development [2,3]. Recombinant human interferon beta (rIFNβ) is routinely used to control exacerbations in relapsing-remitting multiple sclerosis (MS) [4,5]. Although effective in reducing the number of exacerbations and brain disease activity in some patients, rIFNβ produces no benefit in almost one-half of these patients [6,7]. Furthermore, it is not at all certain how significant its long-term effects on disease progression are. Therapy has been associated with a number of adverse reactions, including flu-like symptoms, transient laboratory abnormalities, menstrual disorders, increased spasticity, and dermal reactions [8].

We generated and analyzed longitudinal patterns of gene expression from interferon beta (IFNβ)–treated patients suffering from MS with the aim of identifying preexisting and drug-induced signatures that would predict or explain the clinical response to the drug.

## Results/Discussion

Fifty-two patients with relapsing-remitting MS were followed for at least 2 y after initiation of therapy with IFNβ. Clinical follow-up included a neurological examination every 3 mo and at the time of relapse. At each visit, a blood sample was obtained by venipuncture. After the 2-y endpoint, patients were classified as either good or poor responders based on strict criteria, as described in Materials and Methods. We measured the expression profile of 70 carefully selected genes from peripheral blood mononuclear cells isolated from each patient at each time point, using one-step kinetic reverse-transcription PCR (Dataset S1). This process offers remarkable sensitivity and specificity and a dynamic range of several orders of magnitude, allowing the comparison of expressed transcripts from many different genes without compromising accuracy. Targets for analysis were selected on the basis of their presumed biological action and included genes coding for type I and II IFN-responsive molecules, cytokine receptors, members of the interferon (IFN) signaling and apoptosis pathways, and several transcription factors involved in immune regulation (Table S1). Altogether, more than 70,000 reactions were carried out. A common inherent prediction performance limitation of most high-throughput gene-expression profiling projects arises from the largely asymmetric expression data matrix obtained as a result of measuring far more genes than samples [9]. Such ill-conditioned data matrices inevitably lead to overfitting of predictive models (among other difficulties), some effects of which can be mitigated by judicious application of various established inverse and regularization schemes [10]. The undesirable properties (i.e., overfitting) of such massively under-determined datasets are largely avoided in this study design because the number of genes measured is commensurable with the numbers of samples.

Using linear discriminant analysis–based integrated Bayesian inference system (IBIS), we were able to detect the gene *MX1* as the single best discriminating variable between samples obtained at baseline (*T* = 0) and at 3 mo after initiation of therapy (*T* = 3) with a classification accuracy of 79% (data not shown). Given that *MX1* is a known marker of IFN bioavailability [11], this result validates our experimental approach as well as our sample handling and processing.

**Figure 1:**
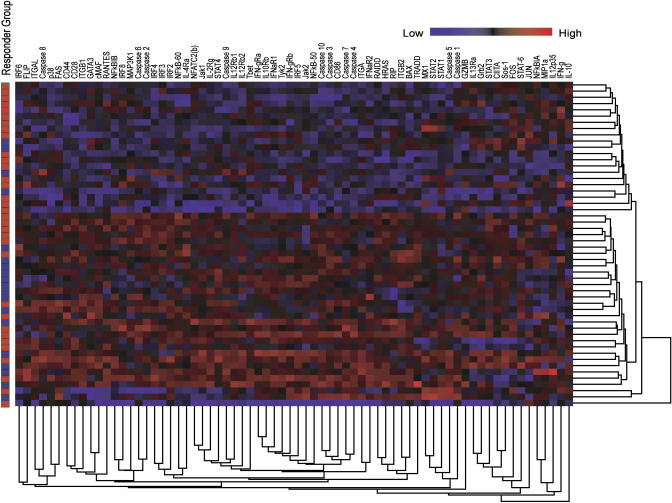
Nonsupervised Two-Way Hierarchical Clustering of Samples at *T* = 0 A clear aggregation of samples cannot be seen by this technique. The first column indicates the type of responder to which each sample belongs (red, good; blue, poor).

To search for expression signatures associated with therapeutic outcome (good or poor responder), we conducted clustering of samples using normalized data for all 70 genes at each time point [12]. Despite applying several different similarity measures and clustering algorithms [13], we did not observe concomitant segregation of samples according to their responder status, with the exception of a few local clusters of small size (Figure 1). This result may suggest that overall differences in gene expression in the two groups of patients, as assessed by conventional similarity measures, are small or negligible. The clustering null results with respect to concomitant class segregation, however, do not rule out the possibility of discovering outcome-predictive combinatorial and nonlinear relationships. To investigate this possibility further, we used quadratic discriminant analysis–based IBIS, implemented for three-dimensional (3D) searches, in the search for highly predictive sets of three genes whose expression at *T* = 0 correlated with a good or poor outcome of therapy at the 2-y endpoint. This process exhaustively carried out sample classification by searching through all 54,740 possible three-gene combinations of 70 genes. Higher-order combinatorial searches beyond combinations of three genes are possible using IBIS and are currently under investigation. However, higher-order predictive variable combinations do require the support of many more samples to prevent overfitting of the model.

We implemented a stringent method for examining the statistical validity of our classification results that consisted in testing the obtained classifier in an independent set of samples not previously “seen” by the program. All of the following statistical analyses were thus performed on split datasets, namely, training (75% of the samples) and test (25%), each reflecting the same relative proportion of classes (63% good and 37% poor responders). We started by conducting 3D IBIS searches using expression data from only the training set and selected the top nine scoring triplets (on the basis of their high prediction accuracy and low mean squared error [MSE] values). For each gene triplet, and using the training data only, a committee of classifiers was built based on an internal cross-validation scheme. Subsequently, the classifiers were used to predict the outcome of an independent test set of samples. Gene triplets were ranked on the basis of the prediction accuracies of the classifiers on this independent test set. We identified nine gene triplets with a predictive accuracy of at least 80% (Table 1). We considered it essential to empirically rule out the chances of fortuitous data splits in the accuracy results obtained from the top-scoring gene triplets.

**Table 1:**
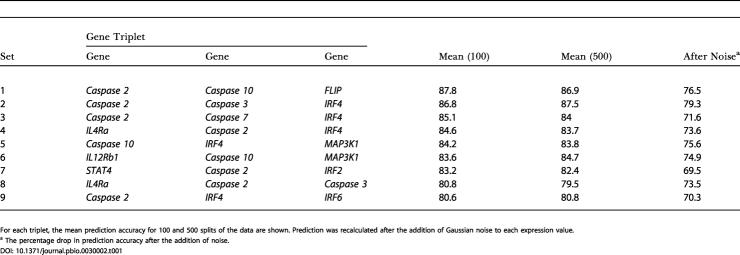
Best-Scoring Predictors of Response to IFNβ at *T* = 0

For each triplet, the mean prediction accuracy for 100 and 500 splits of the data are shown. Prediction was recalculated after the addition of Gaussian noise to each expression value

^a^ The percentage drop in prediction accuracy after the addition of noise

**Figure 2:**
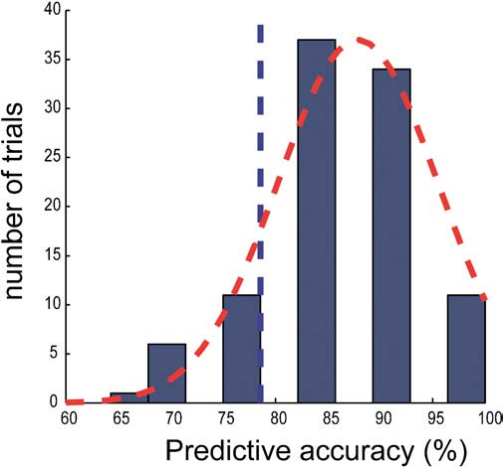
Accuracy Ranges of the Three-Gene Predictive Model of IFNβ Response After the initial data split into training and test sets, using IBIS on the training set only, nine best-performing triplets were identified. The triplet of *Caspase 2, Caspase 10,* and *FLIP* resulted in an accuracy rate of 86% correct prediction on the blind test set resulting from the original split. To minimize the effect of fortuitous initial data division in the accuracy outcome, an extra 100 data splits were performed as a coarse approximation of the possible ranges of accuracies in which this gene triplet could result. A histogram of prediction accuracy over the 100 trials for the gene triplet composed of *Caspase 2, Caspase 10,* and *FLIP* is shown as an example of classification and prediction of response to IFNβ at *T* = 0. A red Gaussian curve encompasses the distribution, where the mean prediction accuracy was 87.9%, with a maximum of 100% (in 11 cases) and a minimum of 64.3% (in two cases). The broken blue line indicates the tenth percentile (78.6%). No major differences were found when we performed the same classification/prediction strategy in 500 random splits of the data.

**Figure 3:**
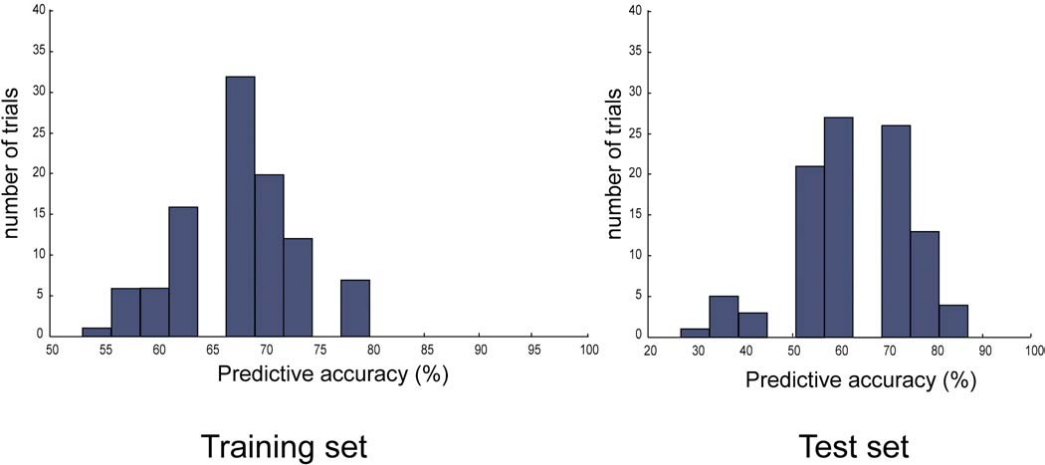
Best-Scoring Gene Triplet by *F*-Test Analysis Notably, as observed with IBIS, *Caspase 10* was also the single best discriminant (*p* = 1.87 × 10^−4^) variable, but the second and third best scoring genes by *F*-test (IL12Rb2, IL4Ra) did not seem to add any significant predictive power. The mean prediction accuracy for the test set of samples was 65.6% (tenth percentile, 57.1%), well below that observed for the triplet derived from IBIS *(Caspase 2, Caspase 10,* and *FLIP)* shown in Figure 2. This suggests that *F*-test could efficiently capture individual linear separators but cannot identify and prioritize the nonlinear combinations of genes discovered by IBIS that ultimately provide the most predictive accuracy and robustness.

Consequently, for the nine top-scoring gene triplets and their corresponding classifiers, we generated 100 random splits and built classifiers for each new resulting training set. Next, we tested how well the classifiers predicted therapeutic outcome in the corresponding test datasets. Figure 2 illustrates the distribution in the prediction accuracies obtained for the triplet composed of *Caspase 2, Caspase 10,* and *FLIP,* which yielded a predictive accuracy of 86% in the original split. The bell-shaped distribution resulting from 100 tests for this triplet displayed a mean accuracy of 87.8% and a tenth percentile of 78.6%, meaning that if the prediction were performed multiple times, in 90% of these instances an accuracy of almost 79% or better would be obtained. This histogram only reflects the range of accuracies obtained, should the initial data split be different. Notably, the genes in the top-scoring triplet were *Caspase 2, Caspase 10,* and *FLIP*—three apoptosis-related molecules. The second-highest-scoring triplet was that of *Caspase 2, Caspase 3,* and *IRF4* (86.8% mean accuracy after 100 splits). Other high-scoring triplets included *IL4Ra* and *MAP3K1,* in addition to other apoptotic molecules (Table 1). When we repeated this experiment with the top three scoring genes, using *F*-test, the obtained mean accuracy was 64% (tenth percentile at 50%) (Figure 3).

**Figure 4:**
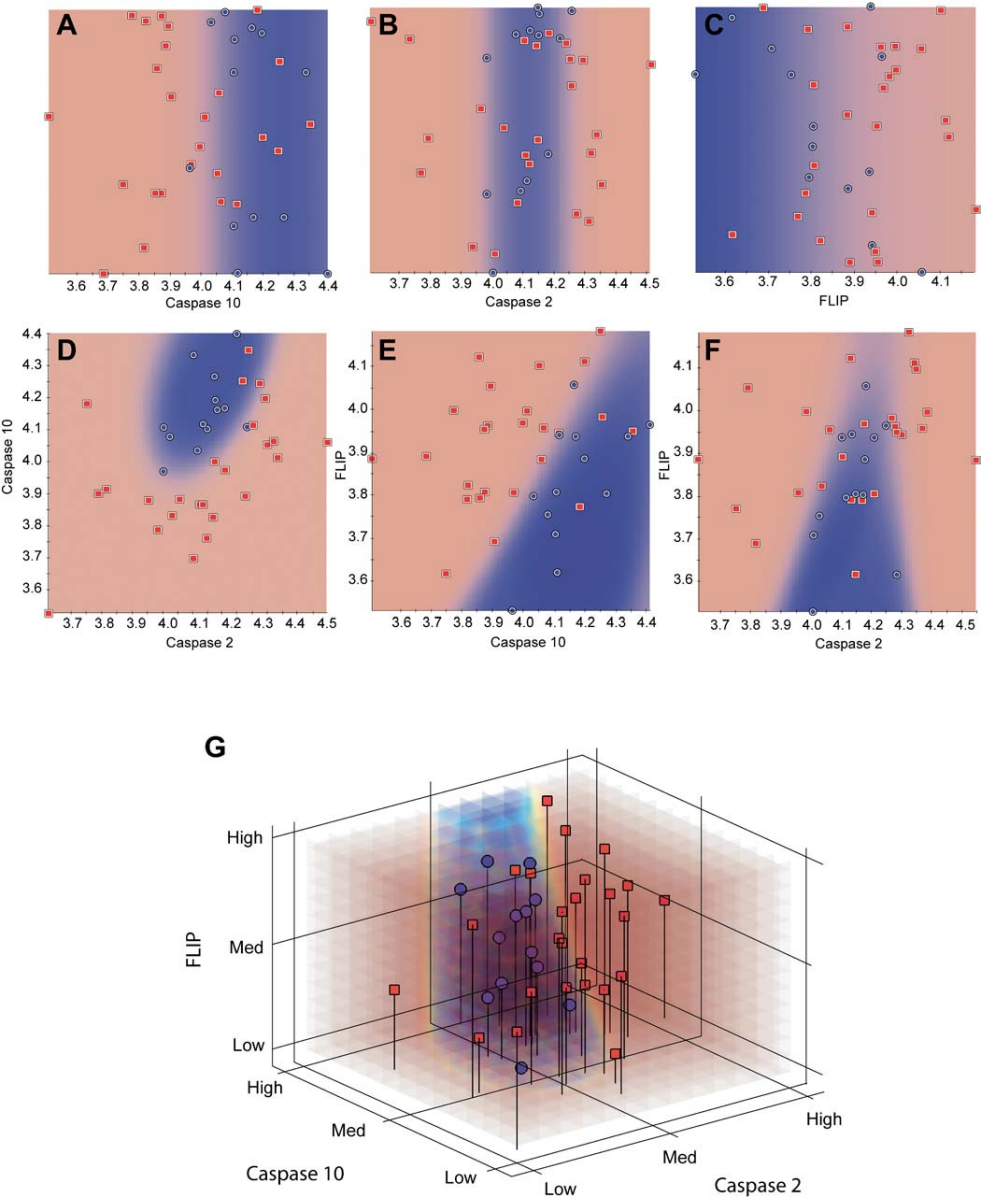
Training Dataset Performance of the Three Genes from the Top Predictive Model of IFNβ Response One-, two-, and three-dimensional IBIS searches were conducted independently on the same training dataset. Each chart shows a two-colored background, corresponding to regions predictive of good response (red) and poor response (blue). Each colored dot corresponds to an individual sample (red, good responder; blue, poor responder). (A–C) One-dimensional IBIS predictive models. High values of *Caspase 10* are associated with poor response according to a linear relationship. In contrast, *Caspase 2* levels are associated with poor response at intermediate values, suggesting a nonlinear relationship. *FLIP* expression is associated with good responders at low values, again depicting a linear relationship. The highest cross-validation accuracy score for a single gene predictor was 73% *(Caspase 10)*. (D–F) Two-dimensional IBIS predictive models. Each of the three possible pairs of this classifier was tested. Linear and nonlinear combinatorial predictive relationships were revealed, specifically, a nonlinear predictive relationship associating poor response with high values of *Caspase 10* and intermediate values of *Caspase 2,* a nonlinear relationship associating good response with high values of *FLIP* and either low or high (but notintermediate) values of *Caspase 2,* and a linear relationship associating poor response with low values of *FLIP* and high values of *Caspase 10*. The highest cross-validation score was obtained for the *Caspase 2/Caspase 10* pair according to a nonlinear, quadratic distribution (85% accuracy). (G) Three-dimensional IBIS predictive model. The shapes identified in the 1D and 2D distributions were optimized by the 3D model, providing a better separation of good and poor responders.

**Figure 5:**
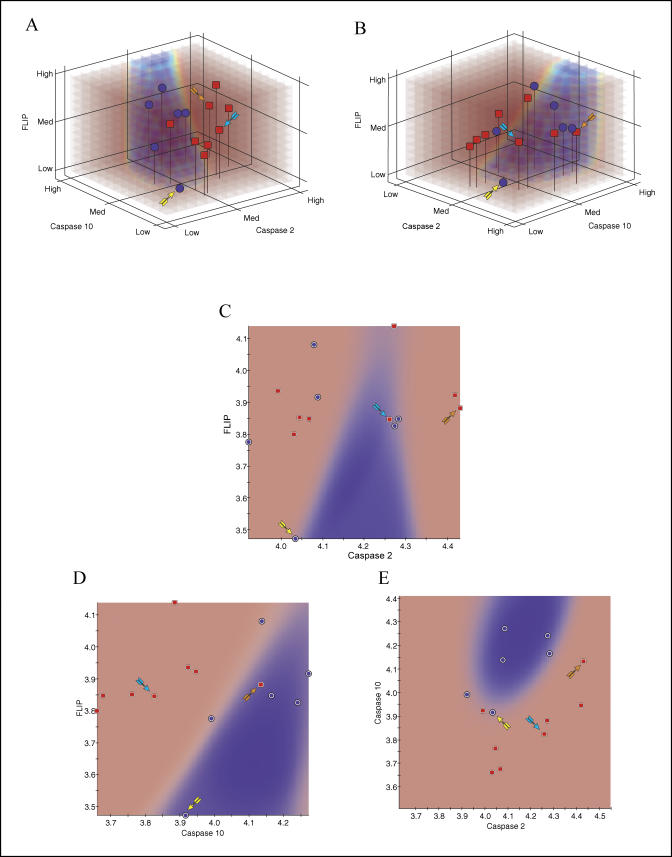
Test Dataset Performance of the Top Three-Gene Predictive Model of IFNβ Response The same probability model generated from the training dataset (see Figure 4G) provides the background shading of volumes predictive of good response (red) and poor response (blue). Three samples are identified with arrows and followed along different graphical representations. (A and B) The two rotations of the full 3D model show that all good responder samples are correctly classified. (C) Projection of full model onto one of the possible 2D surfaces is provided as an aid to visualization. (D–F) Two-dimensional IBIS predictive models. Three samples are identified with arrows and followed along different graphical representations. If prediction was performed in only two dimensions, a higher number of misclassifications would have occurred. For example, the 2D model built using only *Caspase 2/FLIP* (D), could not resolve the good responding sample identified by a cyan arrow, whereas it correctly resolves the good responding sample shown by the orange arrow. The model built using *Caspase 10/FLIP* (E), in contrast, acts oppositely and can resolve the good responding sample shown by the cyan arrow and not the sample shown by the orange arrow. Both these sample are correctly resolved the 2D model built using *Caspase 2/Caspase 10* (F); however, this model is unable to resolve the poor responding sample identified by the yellow arrow, whereas one of the previous models (E) was able to do this. As demonstrated in the full 3D model view from (A) and (B), as well as the projection of model (C), all the labeled poor and good responding patients are correctly classified. Although 2D models show high predictive capabilities, all three genes are needed to increase the classification accuracy of the IBIS model.

In Figure 4, the predictive capability of the best-scoring triplet (*Caspase 2, Caspase 10,* and *FLIP;* 3D model) was compared with those obtained with the single-gene (1D) and gene-pair (2D) models. We observed that the classification accuracy improves as more genes are added to the classifier. We next plotted the samples of a test dataset (25% of samples) on the predictive probability model shown in Figure 4G and compared the performance of the 3D IBIS model to those of the individual 2D models (Figure 5). Overall, the 2D projections of the 3D predictive model show that the *Caspase 2*/*Caspase 10* and *Caspase 10/FLIP* gene pairs show significant predictive capability, but that all three genes are required to provide the highest level of model accuracy and robustness.

To validate the specificity and predictive capability of the top-scoring gene triplet (for the good and poor responding classes) and its associated classifiers, we examined the model exhibiting the best performance on a “default” expression dataset. This null dataset was built keeping the original gene expression data and randomly permuting the class labels of the outcomes 1,000 times (keeping the same counts of good and poor responding patients as were in the original dataset). The prediction accuracies for all the gene triplets obtained with this dataset dropped dramatically as the means ranged from 49.2% to 53.6% (data not shown), emphasizing the specificity of the classifiers. In addition, for the top-scoring gene triplet (for good and poor responder classes), we calculated the probability of achieving, under the null hypothesis, an equal or better accuracy than that obtained in the original prediction (86%), as previously described [14]. This achieved significance level was 0.009, suggesting that it is very unlikely that the prediction accuracies observed for this classifier are caused by chance.

Finally, we tested the robustness of each of these gene sets as predictors of IFNβ response by simulating experimental measurement error. To accomplish this, we first calculated the standard deviation of the expression measurements for all genes as an estimation of the overall experimental error. We then added a fixed amount of Gaussian noise corresponding to one standard deviation (taken from 20 random deviations) to each expression value and repeated the classification/prediction in 30 different splits of the data (a total of 600 tests). Notably, the mean drop in predictive accuracy after the addition of noise was less than 10%, denoting a significant tolerance to reasonable measurement errors (Table 1).

Because all the patients in this study were systematically followed up for a period of 2 y, we were able to perform a longitudinal analysis. Using a repeated-measures analysis of variance (ANOVA), we searched for genes with significantly different expression patterns based on models that tested for responder effect, time effect, and interaction effect (time × response).

**Figure 6:**
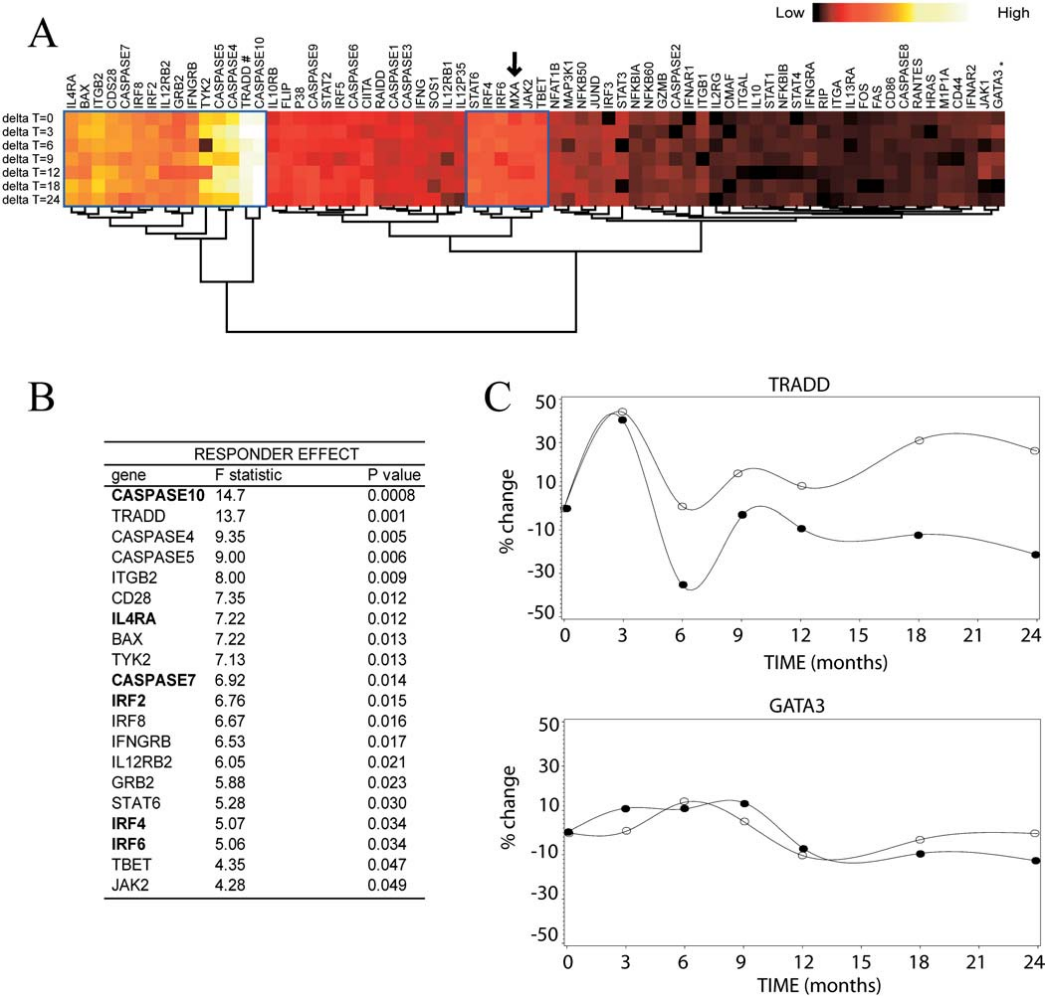
Characteristic Gene Expression Profiles of Good and Poor Responders to IFNβ over Time (A) An unsupervised hierarchical clustering representation of the weighted difference between the average expression of good and poor responders. For each gene, the obtained differences were log normalized and multiplied by the *F*-statistic from an ANOVA (responder effect) run previously (shown in [B]). The “heat” colored bar represents the absolute value of this difference. With the exception of *MX1* (indicated by an arrow), all genes showing a significant difference in expression between the two groups of patients were automatically arranged in only two clusters (framed in blue). (B) List of all genes showing a significant responder effect along with their *F*-statistic and *p*-values. Genes that were part of any triplet showing more than 80% prediction accuracy at *T* = 0 are shown in bold. (C) A continuous representation of the longitudinal average expression of two representative genes for good (^) and poor (•) responders. *TRADD* shows two widely parallel curves, indicative of a significant difference in the expression averages, correlating with its profile (#) observed in the clustering shown in (A). In contrast, *GATA* *3* displays two almost overlapping curves, consistent with its shading (*) in the clustering in (A).

**Figure 7:**
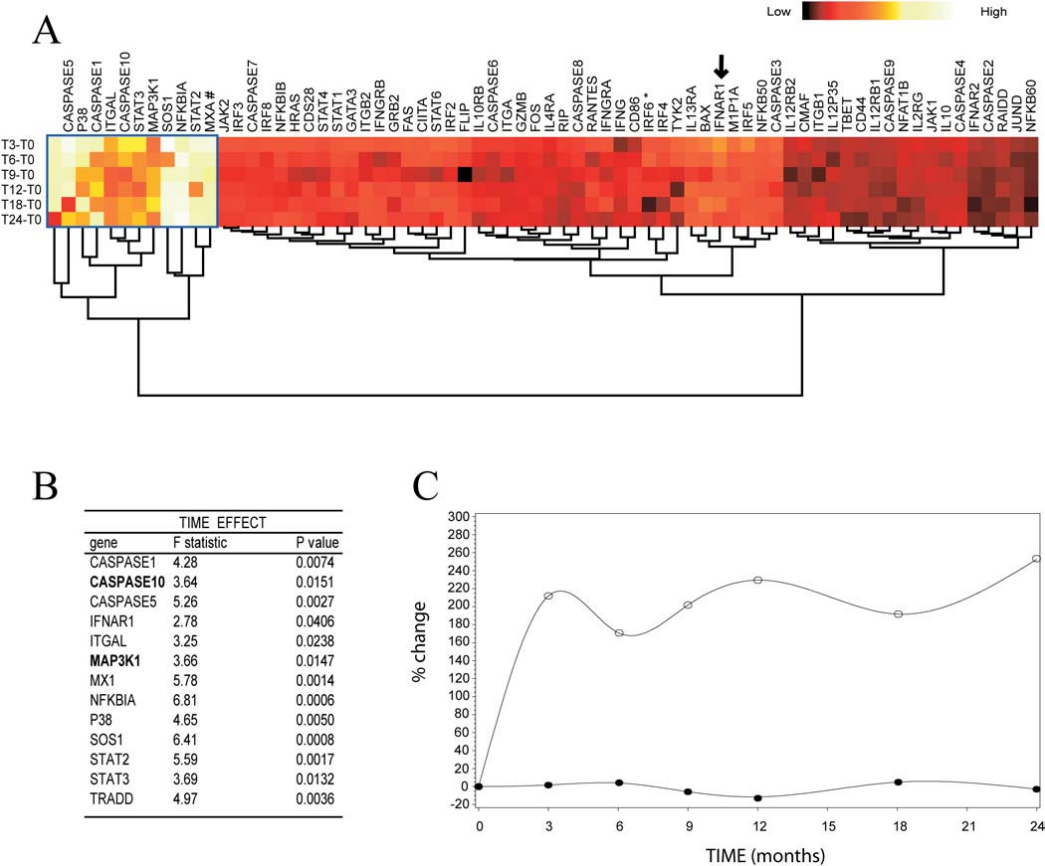
IFNβ-Induced Changes in Gene Expression over Time (A) An unsupervised hierarchical clustering representation of the weighted difference in gene expression at each time point versus baseline. For each gene, the obtained differences were log normalized and multiplied by the *F*-statistic from an ANOVA (time effect) run previously (shown in [B]). The “heat” colored bar represents the absolute value of this difference. With the exception of *IFNAR1* (arrow), all genes showing a significantly different expression in at least one time point with respect to baseline were arranged in the same cluster (framed in blue). (B) List of all genes showing a significant time effect along with their *F*-statistic and *p*-values. Genes that were part of any triplet showing more than 80% prediction accuracy at *T* = 0 are in bold. (C) A continuous representation of the longitudinal average expression of two representative genes over all samples. *MX1* (^) shows a marked departure from *T* = 0 and remains elevated for the rest of the observed period. This correlates well with the shading (#) displayed in the clustering shown in (A). In contrast, *IRF6* (•) displays an almost flat curve, consistent with its color in the clustering (*).

Significant responder effect for 20 genes (Figure 6) and significant time effect for 13 genes were detected (Figure 7). Interestingly, six of the genes that showed statistically significant differences between good and poor responders, *IRF4* (*p* = 0.03), *IL4Ra* (*p* = 0.01), *Caspase 10* (*p* = 0.0008), *Caspase 7* (*p* = 0.01), *IRF2* (*p* = 0.02), and *IRF6* (*p* = 0.03) are among the 12 genes that best predict response at *T* = 0 (shown in bold in Figure 7B). A pattern consistent with increased apoptosis (five members of the Caspase family of proteins, *TRADD,* and *BAX*) was observed for the poor responders.

Although we successfully identified informative, combinatorial relationships, establishing the causality of the association between gene expression and outcome to therapy is beyond of the scope of this work, and these genes are therefore considered surrogate markers. Moreover, although extensive in vivo and in vitro experiments have been conducted, the full mechanism of action of IFNβ in MS remains unknown.

Transcription profiling experiments have involved IFNβ in the regulation of apoptosis in both cancer and MS [15,16,17,18]. Induction of programmed cell death could lead to a reduction in the number of activated lymphocytes, macrophages, and monocyte-derived dendritic cells—all key components of the pathogenic process leading to tissue damage in MS [19,20,21]. However, increased levels of some anti-apoptotic molecules have also been observed in IFNβ-treated MS patients, possibly reflecting a compensatory mechanism [16]. Furthermore, even the inhibition of activated T cell apoptosis in response to IFNα and IFNβ has been reported [22]. Our finding of increased apoptosis in poor responders does not support the hypothesis of programmed cell death as a primary therapeutic mechanism for IFNβ. We hypothesize that a net increase in pro-apoptotic transcripts in peripheral blood mononuclear cells from poor responders could be reflecting undesired elimination of certain regulatory cell populations that are much needed to maintain a homeostatic balance.

Other differentially expressed transcripts included *IRF4,* a gene essential for mature T and B lymphocyte function and homeostasis, and a transcription factor with dual function (activator/repressor) that regulates transcription of *IL4* through physical interaction with *NFATc2* [23]. Remarkably, *IRF4* is a repressor of other IFN-induced genes [24], an observation consistent with the elevated expression of *IRF4* observed in the poor responders before initiation of therapy.

As expected, the gene *MX1* showed a significant time effect independent of clinical response (*p* = 0.01). This result is in agreement with previous findings indicating substantial *MX1* upregulation in response to type I IFNs [25]. Interestingly, upregulation of *MX1,* which occurs minutes after IFN stimulation [26], is sustained over at least 2 y, spanning several orders of magnitude of time units. This also correlates well with our results identifying *MX1* as the best single classifier for samples from patients before (*T* = 0) and after (*T* = 3) initiation of therapy. In fact, as Figure 7 illustrates, most of the significance for the time effect in *MX1* comes from the difference between *T* = 0 and *T* = 3.

Also of interest, a significant time effect (but not responder effect) was observed for *IFNAR1* and *STAT2* (Figure 7B). Because *IFNAR1* is a subunit of the heterodimeric type I IFN receptor and *STAT2* is a critical component of the DNA binding complex ISGF3a (which regulates the expression of IFN-responsive genes), their upregulation on administration of rIFNβ is likely related to mechanistic aspects of IFN signaling. Our results suggest that poor response is associated with downstream signaling events rather than deficient recognition or metabolism of the drug. Our previous finding that IFN receptor polymorphisms do not affect therapeutic response in this same set of patients partially supports this hypothesis [27]. Only two genes with significant time effects, *Caspase 10* (*p* = 0.01) and *MAP3K1* (*p* = 0.01) were part of any predictor set (Figure 7B). In addition, *MAP3K1* also showed a significant interaction effect (*p* = 0.05; data not shown). These results highlight the involvement of these genes in the response to IFN both at *T* = 0 and once therapy has started.

Here we combined large-scale, function-oriented gene expression with advanced data mining to identify a set of markers that accurately and robustly predict the response to rIFNβ therapy. Although larger, prospective studies are needed to confirm these findings, our results suggest that the underlying gene activity profile of an individual at the verge of therapy harbors sufficient information to allow investigators to estimate the chances of experiencing satisfactory therapeutic effects. As analytical tools to predict clinical outcomes based on molecular evidence evolve, these types of studies are likely to become a substantial aid to the physician, taking the paradigm of personalized medicine one step further.

## Materials and Methods

### 

#### Patients and samples

All studies were approved by the respective Committees of Human Research at Hospital Vall d'Hebron, Barcelona, Spain, and the University of California, San Francisco, United States. Informed consent was obtained for all study participants. All patients were examined by a trained neurologist at the CNI Unit, Vall d'Hebron Hospital. Inclusion criteria for this study were clinically definite MS (Poser's criteria), disease in relapsing-remitting phase, age between 18 and 65 y, recorded history of at least two clearly identified relapses within the preceding 24 mo, and expanded disability status scale between zero and 5.5 (inclusive). Detailed information about clinical aspects of these patients has been recently reported elsewhere [6].

Patients were categorized as good responders (*n* = 33) if they experienced a total suppression of relapses and no increase in the expanded disability status scale after a 2-y follow-up period. Poor responders (*n* = 19) were defined as having suffered two or more relapses or having a confirmed increase of one point in the expanded disability status scale score. Patients with intermediate phenotypes were excluded from this study. Blood specimens were taken following institutional guidelines at approximately the same time of the day just before the administration of the first dose of rIFNβ and every 3 mo thereafter during the neurological examination, with the exception of *T* = 15 and *T* = 21 mo. Altogether, 336 samples were tested (an average of 6.5 time points along 2 y for each individual). Immediately after being drawn, all blood samples were spun over Ficoll-Paque (Amersham Biosciences, Piscataway, New Jersey, United States) gradients to enrich the sample for mononuclear cells. After three washes with PBS, aliquots of 5 × 10^6^ cells were frozen in RPMI1640 containing 20% DMSO and 20% fetal calf serum.

#### RNA purification, quantitation, and handling

Peripheral blood mononuclear cells were thawed at 37 °C for 1 min, and RNA lysis buffer was added immediately. RNA was purified with the Strataprep kit (Stratagene, La Jolla, California, United States) and finally resuspended in nuclease-free water (Promega, Madison, Wisconsin, United States). One-microliter RNA aliquots were subjected to fluorescence-based quantitation (in duplicate) using the Ribogreen reagent (Molecular Probes, Eugene, Oregon, United States) in a Spectra Max Gemini fluorometer (Molecular Devices, Sunnyvale, California, United States). Samples were diluted to 1 ng μl^−1^ using nuclease-free water, and 5 μl was aliquoted in triplicates into 384-well plates using a Multiprobe II liquid-handling instrument (PerkinElmer Life and Analytical Sciences, Boston, Massachusetts, United States). Plates were kept frozen at −70 °C until needed.

#### One-step kinetic reverse-transcription PCR

A master mix was prepared essentially as described previously [28], with the addition of 200 μM ROX (Sigma, St. Louis, Missouri, United States), and overlaid on top of each well of a freshly thawed 384-well plate containing 5 ng of RNA in each well. Reactions were performed in triplicate using an ABI 7900 Sequence Detection System (Applied Biosystems, Foster City, California, United States). Positive and negative controls, as well as calibration curves, all in triplicate, were also included in each reaction plate. Total reaction volume was 10 μl. All expression values were calculated by interpolation in a calibration curve spanning five orders of magnitude constructed with an in vitro transcribed clone of GAPDH. The average of each expression measurement was then divided by that of one of the positive controls (thymus RNA) to account for plate-to-plate variability. On the basis of reports addressing the limited utility of normalization [29,30] and of our unpublished observations, we avoided housekeeping gene normalization and used instead RNA content, thus relying on repeated precise fluorescence-based quantitation and highly accurate liquid-handling procedures.

#### Data collection and analysis

A custom Microsoft Excel (Microsoft, Redmond, Washington, United States) worksheet was prepared for handling reaction data import and performing initial statistics. Normalized data were imported as a .csv file into GeneLinker Platinum (Predictive Patterns Software, Kingston, Ontario, Canada) for preprocessing and clustering analysis. Quadratic discriminant analysis–based IBIS implemented for 3D searches was carried out at the School of Computing, Queen's University, Kingston, Ontario, Canada, and at Biosystemix, Sydenham, Ontario, Canada. IBIS is a data-mining algorithm that searches through the gene space for a single gene (or group of genes) that can predict the outcome class (in this case, good and poor response to rIFNβ therapy). This algorithm incorporates a complete 10-fold cross-validation method with several independently trained classifiers to predict an outcome on the basis of a voting scheme (see below). We used MSE and classification accuracy to assess how well the classification predictions matched the true response of the patients to therapy. The top-performing gene triplets were selected on the basis of a mixed threshold for low MSE levels and high accuracy rates.

#### The algorithm

IBIS identifies genes (or gene pairs or groups of genes) that are highly predictive of the outcome based on probability distributions of those genes in different outcome classes. For example, for a given gene *g_i_,* two Gaussian functions are fitted to the distributions of the observed expression levels in good responding and poor responding patients (let us call these fitted distributions *D*_g_ and *D*_p_ for good and poor responding patients, respectively). Our fitted distribution, *D*_g_(*x*), denotes the probability of a patient having an expression level of *g_i_ = x,* given that this patient is a good responder. The fundamental question we are aiming to answer using data-mining methods (here using IBIS particularly) is as follows: what is the probability of a patient being a good responder given the observed expression level of a gene for that patient? Taking advantage of the fitted distributions, a classifier applies Bayes' formula to answer the fundamental question. According to this formula:



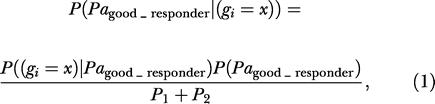



where *P*_1_ *= P*((*g_i_ = x*)*|Pa*_good_responder_)*P*(*Pa*_good_responder_). In fact, *P*((*g_i_ = x*)*|Pa*_good_responder_) is the distribution function fitted to the observed gene expression values of *g_i_* above *(D*_g_ *),* and *P*(*Pa*_good_responder_), the probability of a patient being a good responder, can be easily calculated using the total and good responding patient counts. The term *P*_2_ is strictly analogous to *P*_1_ but applies to poor responders. Therefore, according to equation 1, for a given gene, a comprehensive model is built that predicts the probability of a patient being a good responder for different values of observed expressions of that gene. IBIS searches through all the genes and calculates such models for a single gene or combination of genes, resulting in singular or combinatorial mining of most relevant genes. The probability of a patient being a poor responder can also be calculated in a similar fashion.

To obtain a reliable classifier that is generalizable to all patient samples obtained under similar conditions, the Gaussian distributions and the classifier were only trained on a subsample of the patient data (training set). The results of the classification (i.e., probability of a patient being a good or poor responder), however, were tested on patient samples never seen by the classifier before (test set). This ensures limitation of the classifier overfitting. Further, a complete 10-fold cross-validation scheme was built into the training phase. In IBIS, linear and quadratic classifiers correspond to classifiers built using Gaussian distributions with equal or different covariance matrices, respectively. The prediction results of IBIS are visualized graphically within the observed gene expression space by presenting the probabilities of a patient being a good or poor responder as a background color (see Figures 4 and 5). The red background in the gene space represents a high probability of a patient sample being a good responder if the observed gene expression values are in that range in the gene space. The blue background, similarly, represents a high probability of a patient sample being a poor responder if the observed gene expression values are in that range in the gene space.

Several measures were used to assess how well the calculated probabilities matched the true patient responses to therapy. Two of these measures were MSE and classification accuracy. MSE was calculated as the sum of (response*_i_* ^observed^ − response*_i_* ^expected^)^2^ averaged over all patients. For a given patient, the clinical response determined by the end of the 2-y monitoring period is denoted by response*_i_* ^observed^ and response*_i_* ^expected^ and represents the probability of that patient being a good responder to rIFNβ therapy, using the Bayes' formula above. Classification accuracy simply expresses the percentage of patients that were correctly predicted as being good or poor responders.

#### Classification and prediction procedure

The initial dataset of patients was divided into two parts; namely, a training set with 75% of the samples and a test set with 25% of the samples, each reflecting the same proportion of classes (63% good and 37% poor responders). Only the training set was used for identifying the best predictive gene triplets with the IBIS method, as well as for building the classifier. A committee of classifiers was then generated using a 10-fold cross-validation scheme during training. The training data themselves were divided into ten parts, and each time, a classifier was built using only nine parts of the data. That classifier's predictive capability was determined by its accuracy over the one-tenth of the data withheld. A committee of ten classifiers was assembled from the results of this training stage; this committee was then applied to the test data (which have thus far been hidden from the classifiers). For a patient sample in the test data, each classifier in the committee made a prediction. A majority voting scheme then decided as to which class the sample would be assigned.

Given the initial data split into training and test sets, it was important to rule out the role of fortuitous idiosyncrasies in this split and the resulting accuracy rates. To address this point, we created 100 random splits of the data into training and test subgroups. A committee of classifiers was trained on the training set for each data split, and the accuracies were calculated over the blind test set. A histogram of the test set accuracies was then built, representing the expected ranges of accuracies had the initial data split been different. This histogram is not representative of the estimated or idealized distribution of the accuracies for a gene triplet in a machine learning sense. Rather, it is a coarse approximation of the possible range of gene triplet outcome–prediction accuracies that could be expected.

#### Controlling for false discoveries

To assess the significance and specificity of the top-scoring gene triplets and their corresponding trained committee of classifiers, a null dataset was created by keeping the same expression levels of genes in the dataset and randomly permuting the class labels of the patients 1,000 times (the total count of poor and good responding patients was unchanged). Classifiers were built using the training null data, and accuracies were calculated on the corresponding test sets. The mean of these accuracies for all the top-performing gene triplets was around 50%. The achieved significance level, which represents the probability of achieving accuracy levels better than or equal to that of the nonpermuted classification, was calculated to be 0.009. This value can be considered a significance level, or *p*-value, and indicates the number of times in 1,000 trials for which accuracies of 86% or higher can be achieved under the “no predictive capability” null hypothesis.

Time-series analysis was performed using SAS version 8.0 (SAS Institute, Cary, North Carolina, United States). Permutation analysis and histogram graphic outputs were produced with Matlab (The Mathworks, Natick, Massachusetts, United States).

## Supporting Information

Dataset S1 Raw Expression Dataset Gene expression values for all samples at all time points. This is the raw file from which all analyses were performed.
(491 KB XLS).

Table S1 Target Information Gene names, symbols, and LocusLink and GenBank accession numbers, as well as primer sequences, are listed for all targets.
(160 KB DOC).

### Accession Numbers

The LocusLink (http://www.ncbi.nlm.nih.gov/projects/LocusLink/) accession numbers for the genes discussed in this paper are *BAX* (LLID 581), *Caspase 10* (LLID 843), *Caspase 2* (LLID 835), *Caspase 3* (LLID 836), *Caspase 7* (LLID 840), *FLIP* (LLID 8837), *IFNAR1* (LLID 3454), *IL4* (LLID 3565), *IL4Ra* (LLID 3566), *IRF2* (LLID 3660), *IRF4* (LLID 3662), *IRF6* (LLID 3664), *MAP3K1* (LLID 4214), *MX1* (LLID 4599), *NFATc2* (LLID 4773), *STAT2* (LLID 6773), and *TRADD* (LLID 8717).

## Abbreviations

[number]D: [number] dimensional
ANOVA: analysis of variance
IBIS: integrated Bayesian inference system
IFN: interferon
IFNβ: interferon beta
MS: multiple sclerosis
MSE: mean squared error
rIFNβ: recombinant human interferon beta
