# Supplementary material for: Transcription-Based Prediction of Response to IFNβ Using Supervised Computational Methods
Source: PLoS Biol. 2004 Dec 28;3(1):e2. doi: 10.1371/journal.pbio.0030002 (PMC539058; doi:10.1371/journal.pbio.0030002)
Supplement: Table S1 — Gene names, symbols, and LocusLink and GenBank accession numbers, as well as primer sequences, are listed for all targets. (160 KB DOC). [file pbio.0030002.st001.doc]

**Table S1.** Target information**.**

Gene names, symbols, Locuslink and Genbank accession numbers as well as primer sequences are listed for all targets.

| Symbol | gene description | Locuslink ID | Genebank accession # | Forward primer | Reverse primer |
| --- | --- | --- | --- | --- | --- |
| *BAX* | BCL2-associated X protein | 581 | NM_004324 | GGA CGA ACT GGA CAG TAA | GCC TTG AGC ACC AGT TT |
| *CASP1* | CASPASE1 | 834 | NM_033292 | CGCAAGGTTCGATTT | TGTCCTGGGAAGAGGTAGAA |
| *CASP10* | CASPASE10 | 843 | NM_032977 | GAG CAC ACA GAG GAT TCT A | GCC AGC CTT CAG ATC AA |
| *CASP2* | CASPASE2 | 835 | NM_032982 | CGCGCTCAGACATGATA | ACCTTCCCGATCCTTGATAA |
| *CASP3* | CASPASE3 | 836 | NM_004346 | GGGATCGTTGTAGAAGTCTAA | CGGCCTCCACTGGTATT |
| *CASP4* | CASPASE4 | 837 | NM_001225 | GGGTCATGGCAGACTCTAT | GCTTGAGGGCATCTGTAGATT |
| *CASP5* | CASPASE5 | 838 | NM_004347 | GCT GCT GCC ACC TAA T | GCC AGG AAA GAG GTA GAA A |
| *CASP6* | CASPASE6 | 839 | NM_001226 | GCGAAGGCAATCACAT | CAACCAGGCTGTGACACTT |
| *CASP7* | CASPASE7 | 840 | NM_033338 | CCG TCC CTC TTC AGT AA | GCC CAT ACC TGT CAC TTT A |
| *CASP8* | CASPASE8 | 841 | NM_001228 | AGAGGGAACCTGGTACAT | TCAGGATGGTGAGAATATCAT |
| *CASP9* | CASPASE9 | 842 | NM_001229 | CCA GAC CAG TGG ACA TT | CTC CAT GCT CAG GAT GTA A |
| *CD44* | CD44 | 960 | NM_000610 | GCT CTT GGC TCT CAA CTT A | ACT CCC TTG AGA AGA GAT TGT A |
| *CD86* | CD86 | 942 | NM_006889 | CCA CCT GAA GAA GAT TGT A | TGC TCA CGT CAT CAT CAG TA |
| *CD28* | CD28 | 940 | NM_006139 | ACTGGGTTGGACTGAAAA | AGATTTCCTTGAGTTCCATTCTATAA |
| *CIITA* | MHC class II transactivator | 4261 | NM_000246.1 | ATG CGA CAA CCG TGA TAC AT | AGG TTG CCC AGG AAC TTA C |
| *CMAF* | V-MAF oncogene homolog | 4094 | AF055376 | CTCCCAATGCACTGAA | GTGGGCGTCATTCTTATTAAA |
| *FAS* | FAS antigen | 355 | NM_000043 | GAG GAA GGA TCC AGA TCT A | CTT TCT GCA TGT TTT CTG TAC TT |
| *FLIP* | Flice inhibitory protein | 8837 | NM_003879 | GGA GCA GGG ACA AGT TA | CCA AGC TGT TCC TTA AGT CTT |
| *FOS* | v-fos oncogene homolog | 2353 | NM_005252 | TCTCCAGAAGAAGAAGAGAA | GCCCCCAGTCAGATCAA |
| *GATA3* | GATA binding protein 3 | 2625 | X55037 | GGCATCCAGACCAGAA | GGGTGGTGTGGTCCAA |
| *GRB2* | growth factor receptor-bound protein 2 | 2885 | M96995 | GGGTGGTGAAGTTCAA | CCTGGGGATCAAAGTCAA |
| *GZMB* | Granzyme B | 3002 | NM_004131 | GGT GGC TTC CTG ATA CAA | CGG CTC CTG TTC TTT GAT ATT |
| *HRAS* | v-Ha-ras oncogene homolog | 3265 | NM_005343 | GTG TTT GCC ATC AAC AA | GCA GCC AGG TCA CAC TT |
| *IFNAR1* | interferon (Type I) receptor 1 | 3454 | G03171 | GTGACGCTGTATGTGAGAA | TTCGCAGCATAAATGACAAA |
| *IFNAR2* | interferon (Type I) receptor 2 | 3455 | NM_000874 | GCCTGATTACACAGATGAA | GAAATTGTGTGAGCAACTGAA |
| *IFNG* | Interferon gamma | 3458 | XM_006883 | CGGAAACGATGAAATATACAA | CTCTCCTCTTTCCAATTCTTCAA |
| *IFNGR1* | interferon gamma receptor 1 | 3459 | J03143 | AGGGTTGGACAAAAAGAA | CCTGCTCGTCTCCATTTACAA |
| *IFNGR2* | interferon gamma receptor 2 | 3460 | U05877 | GCATCCCATTACAGATAGAA | CTTTTCCGGAAACGAGATAA |
| *IL10* | Interleukin 10 | 3586 | NM_000572 | GTGGAGCAGGTGAAGAA | CCGATTTTGGAGACCTCTAA |
| *IL10RB* | Interleukin 10 receptor B | 3588 | XM_009733 | TCCCGAAAATGTCAGAA | AATTCAGCCCTGACTCTCAA |
| *IL12A* | IL12P35 | 3592 | NM_000882 | AACCGGATTTTTATAAAACTAA | AGATAGCTCGTCACTCTGTCAA |
| *IL12RB1* | interleukin 12 receptor, beta 1 | 3594 | NM_005535 | CGACACCCACACAGAA | GCAGAGGCAAAGATGGTAA |
| *IL12RB2* | interleukin 12 receptor, beta 2 | 3595 | XM_001390 | GGCGATGTGACTGTGAA | GGGAAGACCTGTGACTTGAGAA |
| *IL13RA1* | interleukin 13 receptor, alpha 1 | 3597 | AB031393 | GTGCAAATGCTAATGTCAA | CCCAGCCCAGACTGTTAAA |
| *IL2RG* | interleukin 2 receptor, gamma | 3561 | NM_000206 | GCCACACAGATGCTAAA | GGCTCCGAACACGAAA |
| *IL4RA* | interleukin 4 receptor, alpha | 3566 | X52425 | CTGGGCTCAGTGCTATAA | GACGGCCAGGATGACAA |
| *IRF2* | interferon regulatory factor 2 | 3660 | NM_002199 | CGG TCC TGA CTT CAA CTA TA | TCA GTG GTC ACC TCT ACA ACT T |
| *IRF3* | interferon regulatory factor 3 | 3661 | NM_001571 | CCA CTG GTG CAT ATG TT | CTC CGG TCC TCT GCT AAA |
| *IRF4* | interferon regulatory factor 4 | 3662 | NM_002460 | CCA GCA GGT TCA CAA CTA | ACG TCA TGG GAC ATT GGT A |
| *IRF5* | interferon regulatory factor 5 | 3663 | NM_002200 | GAG CAG GTG GAA CTC TT | GCA GCT GGT TCG TGT AGA A |
| *IRF6* | interferon regulatory factor 6 | 3664 | NM_006147 | TAC CCC AGG CAC CTA TA | CCT TCC CAC GGT ACT GAA |
| *IRF8* | interferon regulatory factor 8 | 3394 | NM_002163 | GCT GGC AAG CAA GAT TA | GAC CGG TCC GTC ACT T |
| *ITGA4* | Integrin, alpha 4 | 3676 | NM_000885 | GAG CCA AAT CCA AGA GTA A | CCA GCC TTC CAC ATA ACA TA |
| *ITGAL* | Integrin, alpha L | 3683 | NM_002209 | GAA GTG GAG TGC TCC TAT T | CAT CCA GCT GCA GAG TGT AA |
| *ITGB1* | Integrin, beta 1 | 3688 | NM_002211 | CTC AAG CCA GAG GAT ATT | AGA GGT CAA TGG GAT AGT CTT |
| *ITGB2* | Integrin, beta 2 | 3689 | NM_000211 | CCC AGG AAG ACC ACA AT | CCA CCT AGC TTC TTG ACA TT |
| *JAK1* | Janus kinase 1 | 3716 | M64174 | ACTATGCCATGATGAAGAA | CCGCATCCTGGTGAGAA |
| *JAK2* | Janus kinase 2 | 3717 | AF001362 | TACACAGAGAAATTTGAAGTAA | TTTCCCTCTTGACCACTGAA |
| *JUND* | jun D proto-oncogene | 3727 | NM_002228 | ACCCTGCCTTTCCTTTA | GGCGAACCAAGGATTACAA |
| *MIP-1a* | CCL3 | 6348 | NM_002983 | TCA CCT GCT CAG AAT CAT | TGG CTG CTC GTC TCA AA |
| *MAP3K1* | mitogen-activated protein kinase kinase kinase 1 | 4214 | XM_042066 | GTG GGA ACT GGA ACT TTA | GCT CCC AAC ATC CTA ATG AT |
| *MXA* | myxovirus (influenza virus) resistance 1 | 4599 | NM_002462 | GTGGACAAAGGAACTGAA | CGGCACTTGACAATCATGTAA |
| *NFATc2* | nuclear factor of activated T-cells | 4773 | U43341 | AAGCCACGGTGGATAA | TTCCCATTGATGACGTAGAA |
| *NFKB1* | nuclear factor (p105) | 4790 | M58603 | ACGCCATCTATGACAGTAAA | ACAATGGCAAATTGTCTATGAA |
| *NFKB2* | nuclear factor (p49/p100) | 4791 | U09609 | GTGCCTCCAGTGAGAA | AGGACACCCAGGTTGTTAAA |
| *NFKBIA* | nuclear factor inhibitor, alpha | 4792 | NM_020529 | GGC CAG CTG ACA CTA GAA | GCC CCT TTG CAC TCA TAA |
| *NFKBIB* | nuclear factor inhibitor, beta | 4793 | NM_002503 | CAC TTG GCT GTG ATT CAT | TCA TTC TGC AGG TCC ATG TA |
| *P38* | mitogen-activated protein kinase 1 | 5594 | L35253.. | GAG AAC TGC GGT TAC TTA | ATG GGT CAC CAG ATA CAC AT |
| *RAIDD* | death adaptor molecule | 8738 | NM_003805 | GCC CTA AAG CAT TTG ATA | GAG GGG ATC CCA GTC AAT |
| *RANTES* | chemokine (C-C motif) ligand 5 | 6352 | NM_002985 | CGG GAG TAC ATC AAC TCT | CTG CTG CTG TGT GGT AGA A |
| *RIP* | nucleoporin-like protein RIP | 3267 | NM_003804 | GGC CAA TTC CAA GTC ATA | TCC CAC CAA TCT CCA TAT AA |
| *SOS1* | son of sevenless homolog 1 (Drosophila) | 6654 | L13857 | CAGCTGAAGAGAAAAACAA | GGCTGCATGTTCTCTTCAA |
| *STAT1* | signal transducer and activator of transcription 1 | 6772 | NM_007315 | GTTCGCACTCTGTGTATATAA | CATGGGAAAACTGTCATCATAA |
| *STAT2* | signal transducer and activator of transcription 2 | 6773 | NM_005419 | GCAGGAAACTCTCAATGAA | GCTGCCTCAGGTGAAACAA |
| *STAT3* | signal transducer and activator of transcription 3 | 6774 | L29277 | GCGTCCAGTTCACTACTAA | TGCAGCTCCTCAGTCACAA |
| *STAT4* | signal transducer and activator of transcription 4 | 6775 | L78440 | GGAGGCAGCTTCTAACAA | CACATTCCTCTGTCTTTCTGAA |
| *STAT6* | signal transducer and activator of transcription 6 | 6778 | U16031 | CGACATGGTGACAGAGAA | CGGGGCCAAGTGTGAA |
| *TBET* | T-box 21 | 30009 | AF241243 | GGAAACTAAAGCTCACAAA | AGGGGATGCTGGTGTCAA |
| *TRADD* | TNFRSF1A-associated via death domain | 8717 | NM_003789 | GAT TCT GCC TCA GGT ACT T | CCA GGA CAC CAA AGA TCA A |
| *TYK2* | tyrosine kinase 2 | 7297 | NM_003331 | GTCTGTGCTGAATGTGTAA | TGGAGCTCCCTGTGTCAA |
